# Exercise-Induced Right Ventricular Outflow Tract Tachycardia in a Patient with Isolated Left Ventricular Noncompaction

**DOI:** 10.5402/2011/729040

**Published:** 2011-05-26

**Authors:** Tolga Sinan Güvenç, Erkan İlhan, Ahmet Taha Alper, Mehmet Eren

**Affiliations:** ^1^Kafkas University Faculty of Medicine, Department of Cardiology, 5 Kars, Turkey; ^2^Van Ercis State Hospital, Department of Cardiology, Van, Turkey; ^3^Siyami Ersek Thoracic and Cardiovascular Surgery Center, Training and Research Hospital, Department of Cardiology, Istanbul, Turkey

## Abstract

Isolated left ventricular noncompaction is a hereditary cardiomyopathy in which a variety of supraventricular and ventricular arrhythmias could be observed. We report a patient with exercise-induced ventricular tachycardia with left bundle branch block morphology that had characteristics of an idiopathic ventricular tachycardia who was subsequently diagnosed as left ventricular noncompaction. Successful remission of arrhythmia was ensured after the introduction of oral beta-blocker therapy.

## 1. Introduction

Isolated left ventricular noncompaction (LVNC) is a rare cardiomyopathy with a presumed hereditary origin. Lethal ventricular arrhythmias, which are considered as a result of the disease process, are frequently encountered in these patients, and implantation of an implantable cardioverter-defibrillator (ICD) is usually indicated. Right ventricular outflow tract tachycardia is a benign ventricular tachycardia (VT) that is observed in structurally normal hearts, while VTs caused by structural heart disorders may well originate from this location. In this paper, we describe a 52-years-old patient with coexistent left ventricular noncompaction and ventricular tachycardia which was presumed to originate from right ventricular outflow tract (RVOT), with clinical characteristics similar to idiopathic VT.

## 2. Case Report

**Figure 1:**
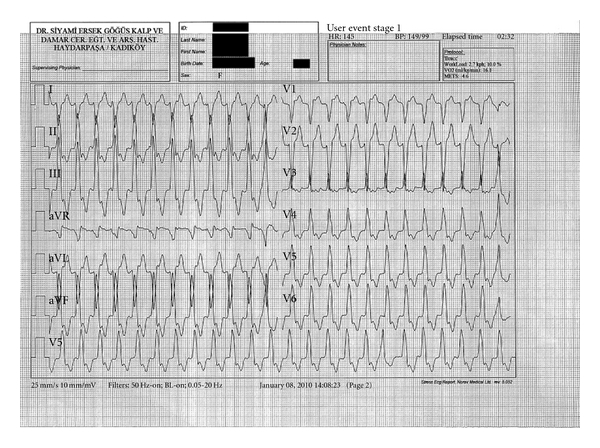
Electrocardiogram taken during maximal exercise. Note the configuration of QRS, with a left bundle branch block pattern, right axis deviation, and positive deflections in leads DII, DIII, and aVF. VT was preceded by single and couplet ventricular extrasystoles with a similar configuration.

**Figure 2:**
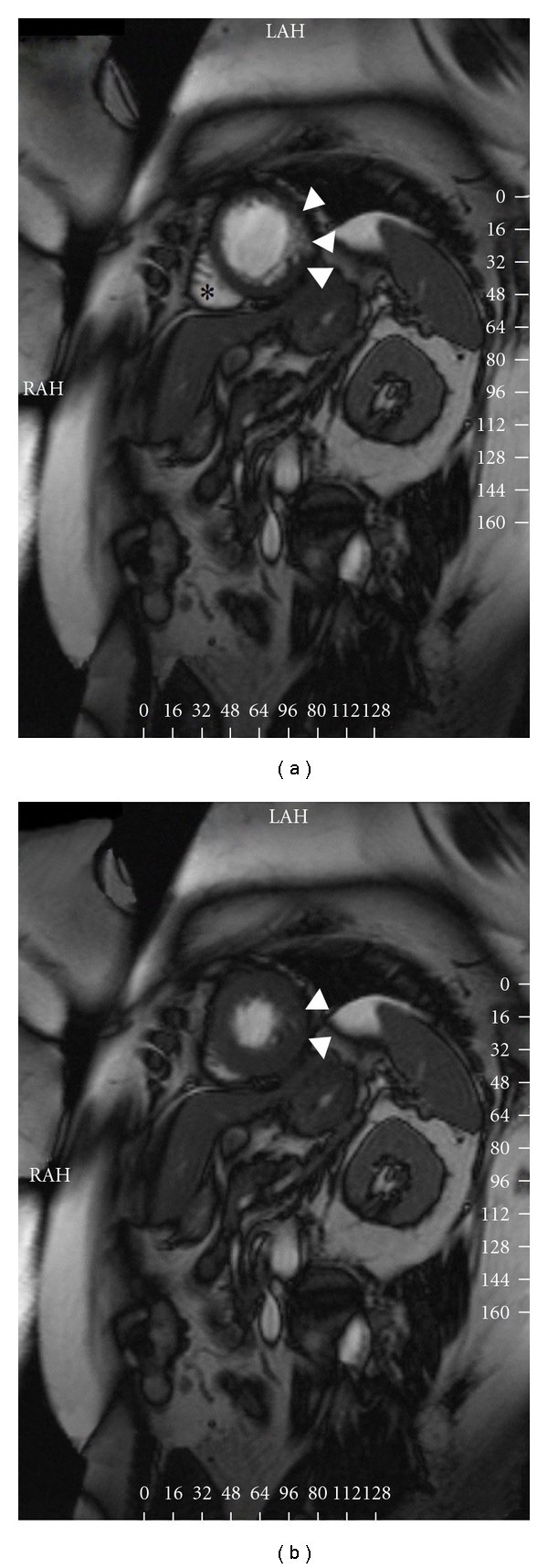
(a) Cardiac MR of the patient taken at diastole. Numerous hypertrabeculated segments with deep intertrabecular recessus separating them are visible in the mid segment of the lateral left ventricle (Arrowheads). Also note that the right ventricle was not affected by the disease process and fibrofatty infiltration was not present (asterisk). (b) Cardiac MR of the patient taken at systole (arrowheads). An endocardial-to- epicardial ratio of 2 in the lateral left ventricular wall was measured in the still frames taken at end-systole.

A 52-years-old female patient admitted to our clinic with chest pain and palpitations occurring during exercise. Her past medical recordings were unremarkable except for mild essential hypertension. No significant findings were noted during physical examination. Her ECG was normal with a sinus rhythm and a normal QRS contour. As her symptoms appeared during exercise, an exercise ECG was performed, and during test, numerous ventricular extrasystoles with a LBBB pattern and positive QRS in leads DII-DIII-aVF ensued and succeeded by sustained ventricular tachycardia with a similar configuration (Figure 1). After application of 6 mg adenosine, her rhythm reverted back to sinus rhythm. Transthoracic echocardiography revealed a small-to-moderate sized noncompacted zone in mid- and inferior walls of left ventricle, along with hypokinesia of the aforementioned areas. Left ventricular ejection fraction was calculated as 45%. To ascertain the diagnosis, cardiac magnetic resonance imaging (CMR) was ordered. CMR revealed similar results with an endocardial-epicardial myocardial ratio of 2 (Figure 2). No features suggesting an alternative diagnosis such as arrhythmogenic right ventricular dysplasia were noted. Coronary angiography, which was performed to rule out ischemic heart disease, revealed normal coronaries. As she had a structural heart disease, an electrophysiologic study (EPS) was performed, while neither ventricular nor supraventricular tachycardia could be stimulated with EPS. As the nature of VT appeared as benign, with subtle symptoms observed during arrhythmia, medical therapy with bisoprolol was initiated and the exercise test was repeated one week later. The latter test was found as unremarkable except scarce ventricular extrasystoles. As beta-blocker therapy seemed to control arrhythmia, ICD implantation was not considered. Six months after her initial admission, she had no complaints in her repeat visit and ECG was normal.

## 3. Discussion

Left ventricular noncompaction is a rare disorder characterized by hypertrabeculation of myocardium accompanied by blood-filled deep intertrabecular recessus separating them [1]. Main manifestations include dyspnea due to left ventricular dysfunction, palpitations and syncope due to supraventricular and ventricular arrhythmias, and recurrent cerebrovascular accidents due to embolization [2, 3]. A variety of supraventricular and ventricular arrhythmias accompanying isolated left ventricular myocardium were reported in literature. Ventricular tachyarrhythmias had a reported incidence of 41% in a series [2]. Sustained monomorphic ventricular tachycardias with RBBB [4] or LBBB morphologies [5] or alternating morphologies [6] were reported previously, as well as polymorphic VT or VF [7]. Echocardiographic criteria for LVNC include blood-filled deep intertrabecular recessus separating hypertrabeculated noncompacted myocardium, with an endocardial to epicardial ratio of 2 or more measured at end-systole [1]. Cardiac MRI is further suggested as a diagnostic modality to confirm the presence of LVNC. We established the diagnosis of LVNC in inferolateral and lateral walls of left ventricle with echocardiography and further supported the diagnosis with cardiac MRI (Figure 2). 

Idiopathic VTs are commonly benign entities which are seen in, by definition, structurally normal hearts. RVOT appears as the most common focus of these arrhythmias. Beta blocking agents are usually employed as the first-line therapy, while catheter ablation remains a definite form of therapy. ICD implantation is not usually undertaken in these patients. On the other hand, VTs seen during the course of LVNC usually necessitate implantation of an ICD, as these patients carry a high risk for sudden cardiac death [8]. In our patient, both the ECG (Figure 1) and clinical features of tachycardia had characteristics of an idiopathic VT, although the patient had structural heart disease. The lack of syncope and hemodynamic deterioration during tachycardia was considered as benign features, and ICD implantation was judged not necessary as VT could be controlled with oral beta-blocker therapy.

Another widely appreciated cause of VT with LBBB morphology is arrhythmogenic right ventricular dysplasia. However, LVNC should also be considered along with ARVD in the differential diagnosis of VTs with a right ventricular origin. In a series, all patients initially identified as ARVD based on echocardiography were subsequently reevaluated due to irrelevant clinical findings and diagnosed as isolated LVNC [5]. Of these patients 3 had VT episodes with LBBB morphology. To rule out the diagnosis of ARVD, we performed cardiac MR in our patient and found right ventricle had normal diameters and function, while fatty infiltration was not present (Figure 2).

In conclusion, we observed a RVOT VT mimicking idiopathic VT in a patient with LVNC and considered a thorough investigation is required to exclude LVNC along with ARVD in VTs with LBBB morphology. While an ICD is deemed necessary for LVNC patients who experienced an episode of VT, therapy should be individualized as LVNC is still an enigmatic disease and it is not known whether all VTs are a consequence of the disorder or not.

##  Conflict of interests

The authors declare that they have no commercial associations or sources of support that might pose a conflict of interest.
